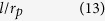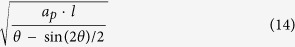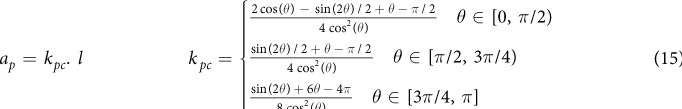# Nanostructure Formation by controlled dewetting on patterned substrates: A combined theoretical, modeling and experimental study

**DOI:** 10.1038/srep32398

**Published:** 2016-09-01

**Authors:** Liang-Xing Lu, Ying-Min Wang, Bharathi Madurai Srinivasan, Mohamed Asbahi, Joel K. W. Yang, Yong-Wei Zhang

**Affiliations:** 1 Institute of High Performance Computing, A*STAR, Singapore 138632, Singapore; 2 Institute of Materials and Research Engineering, A*STAR, Singapore 117602, Singapore; 3 Singapore University of Technology and Design, Singapore 138682, Singapore

## Abstract

We perform systematic two-dimensional energetic analysis to study the stability of various nanostructures formed by dewetting solid films deposited on patterned substrates. Our analytical results show that by controlling system parameters such as the substrate surface pattern, film thickness and wetting angle, a variety of equilibrium nanostructures can be obtained. Phase diagrams are presented to show the complex relations between these system parameters and various nanostructure morphologies. We further carry out both phase field simulations and dewetting experiments to validate the analytically derived phase diagrams. Good agreements between the results from our energetic analyses and those from our phase field simulations and experiments verify our analysis. Hence, the phase diagrams presented here provide guidelines for using solid-state dewetting as a tool to achieve various nanostructures.

Solid state dewetting (SSD) on a patterned substrate has received renewed attention recently, due to its ability to produce nanostructures with controllable sizes and locations. The SSD technique has typically been used to fabricate nanodot arrays with diameter from tens to hundreds nanometers, which are important elements in a variety of nanodots-based applications, including high-density magnetic recording media^1^,^2^, solar cells^3^,^4^, catalysts for nanowire growth^5^,^6^, and many plasmonic devices, such as plasmon-resonance wave guides^7^,^8^, plasmon-enhanced photovoltaic devices^9^,^10^, plasmonic LEDs^11^,^12^, and biosensors^13^. Our recent work^14^ showed that SSD can also be used to fabricate nano-aperture arrays with high yield and at low costs. The diameter of nano-aperture obtained by SSD can be controllable down to 10 nanometers with an aspect ratio of up to 1:5, which makes it a great candidate in the application of nanopore-based single molecule sensing^15^,^16^, x-ray diffraction^17^,^18^, and also plasmonic devices^19^,^20^. Besides nanodot and nano-aperture arrays, Petersen & Mayr^21^ showed that by use of ripple-patterned substrates (pseudo-3D structure for which the 3^rd^ dimension is infinite), SSD can also be used to fabricate nanowire or nanorod arrays, which could be used in nanowire-based applications^22^,^23^,^24^,^25^. In all these demonstrations of SSD, the film thickness and the geometry of the substrate exert great influence on the nanostructure morphology obtained. For example, in the production of nanodot arrays with templated substrates, regular arrays of nanodots are only obtained with an optimal thickness of deposited film. A very thin or thick film usually forms random distributed nanodots even with periodic templates^26^,^27^,^28^. A systematic theoretical analysis that establishes the comprehensive relationships between the system parameters and the resultant nanostructures is crucial in order to fully exploit the potential of the SSD technique for nanofabrication. However, an analytical model to lend these critical physical insights and deep understanding of pattern formation by SSD on topographical substrates is currently lacking. In order to analytically examine the SSD processes on a patterned substrate, one needs to explicitly parameterize the geometry of the formed surface patterns. This is challenging for 3D models for which very few substrate geometries with high symmetries would allow such explicit construction. Examples of these geometries include pillar and pit arrays, where analytical solutions are intractable^29^,^30^

In contrast to the lack of theoretical understanding for SSD, the morphological evolution of liquid wetting layers on chemically^31^,^32^,^33^,^34^,^35^,^36^,^37^,^38^,^39^,^40^,^41^,^42^ or topographically^41^,^42^,^43^,^44^,^45^,^46^,^47^,^48^ structured substrates has been studied extensively and detailed morphological phase diagrams have also been given^31^,^32^,^33^,^35^. In general, there are mainly two types of liquid wetting processes. For the first one, a very thin wetting layer is present on the substrate initially. Subsequently, through deposition or condensation, small liquid droplets start to nucleate, and then grow through coarsening, merging and filling. This process continues as long as the condensation goes on. For the second one, a solid polymer film with a uniform thickness is pre-deposited on a substrate. Subsequently, when the temperature is raised above glass transition temperature, the liquid layer dewets via convective motion. The second type process is very similar with SSD, except that in SSD, even if the temperature is raised, the film is still in solid state and the dewetting process occurs still via diffusion rather than fluid convection. Same as SSD, there is also lack of detailed analysis for this type of process. The first type process, as we will show later, is very different from SSD, and they generally exhibit different dewetting behaviors. In addition, to promote dewetting in SSD, the wetting angle between the solid film and substrate is chosen to be larger than 90°. In contrast, most of previous studies on liquid wetting/dewetting processes were based on the small wetting angle assumption^34^,^35^,^36^,^38^,^40^,^41^,^46^, which is required by the thin-film approximation of the Navier-Stokes equation. As a result, the morphological evolution with a large wetting angle (>90°) has not been fully explored yet, which, as will be shown later, is quite different from that with a small wetting angle (≤90°).

At the nanoscale, the gravity effect is small, and thus can be safely ignored. From the energetic point of view, when applying the macroscopic theory to the nanoscale, we need to consider an additional energy term, i.e., the line tension of the three-phase contact line. Whether to consider this term or not depends on a length scale, which is given by the contact line energy vs. the surface energy. Since for solids, the ratio of the contact line energy vs. the surface energy is about 1 nm (based on typical values of 1 J/m^2^ and 10^−9^ N for surface energy and line tension, respectively). This critical size is much smaller than the feature sizes (>10 nm) in the solid dewetting system. Hence, in our energetic analysis, we can safely ignore this line tension term.

Here, we present an analytical model and an in depth analysis of SSD with pseudo-3D (2D) template geometries. These geometries are of practical interest, for example, in ref. 21, the authors used ripple-patterned substrates to fabricate nanowire and nanorod arrays. Similar chemically line-patterned substrates were also used in many liquid wetting layers, for example in refs 32,34,39,40. In our method, we first perform 2D energetic analysis to find the equilibrium configurations for films deposited on single mesa and pit with rectangular cross section separately. Then, we analyze the inter-diffusion of film atoms deposited on regularly arranged mesas and pits to obtain the resulting phase diagrams. Our results show that by controlling substrate geometries, film thickness and wetting angle, a rich variety of ordered nanostructures can be controllably obtained. We further validate our energetic analysis with phase field simulations and experimental data. In addition to lending deep insights into our understanding of the patterned SSD technique, our study provides important guidelines towards optimizing the SSD technique for a variety of applications, such as metallic interconnects and gratings.

## Results and Discussion

### Template geometry

**Figure 1:**
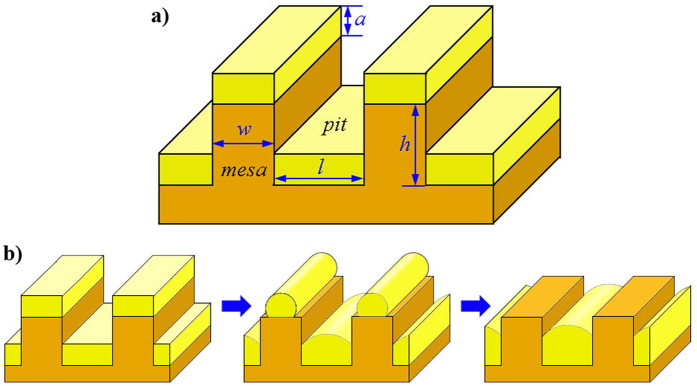
Sketch of the initial model geometry including the as-deposited film and substrate, and the schematic of the dewetting process. (**a**) Depicts the geometry, where *a* is the thickness of the deposited film, *w* is the width of mesas, *l* is the width of pits and *h* is the height of mesas. (**b**) Illustrates the dewetting process, in which a uniformly deposited film together with substrate is heated up to induce dewetting. In this specific case shown here, all the film material on mesa gradually diffuses downwards into the pits.

Figure 1 shows the as-deposited configuration of the template and the film. The template consists of regularly arranged mesas and pits with width of *w* and *l*, respectively. The height of mesas or the depth of pits is *h*. The average (and initial) film thickness is *a*. We assume *h* > *a* to avoid the connection of the films on mesas and pits, which will result in completely different evolution behavior. Furthermore, we normalize all the surface or interface energies by the surface energy of the film. Hence, the normalized surface energy density of the film is *ξ*_12_ ≡ 1. The normalized surface energy density of the substrate is denoted as *ξ*_13_ and the interface energy density between the film and the substrate as *ξ*_23_. The wetting angle according to Young equation is: cos(*θ*) = *ξ*_23_ − *ξ*_13_. Here, the subscripts of 1, 2 and 3 represent the vacuum phase, the deposited film phase and the substrate phase, respectively. Figure 1b shows a typical solid state dewetting process: Initially, a film is deposited on a patterned substrate, and then the film together with the substrate is heated up to induce dewetting via diffusion mechanism. In the specific case shown in Fig. 1b, the film on mesas gradually diffuses downwards into pits.

### Energetic analysis

Given the wetting angle between the film and the substrate as *θ*, the surface area of the bare substrate before deposition as 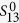, the surface area of the film as *S*_12_ and the interface area as *S*_23_, then the non-dimensionalized total system energy *E* can be expressed as:


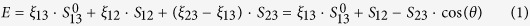


In Equation 1, values of *S*_12_ and *S*_23_ are morphology-dependent, which allows us to identify the most stable morphology by comparing their total interfacial energies. By doing so, we assume that: (1) the film surface keeps its cylindrical shape with a constant mean curvature, similar to previous studies^31^,^32^,^37^; (2) the same wetting angle is chosen for both mesa and pit surfaces, which is different from the cases of chemically patterned substrates^31^,^32^,^33^,^34^,^35^,^36^,^37^,^38^,^39^,^40^,^41^,^42^; (3) the roughness of the substrate is relatively small compared with the film thickness, and hence its influence on the dewetting process can be considered by choosing a proper wetting angle; and (4) there is no chemical reaction or intermixing between the film and substrate.

As described above, the mechanism for morphological evolution in SSD is diffusion, which includes the short-distance diffusion of film atoms along its surface and the long-distance diffusion of film atoms along the sidewalls of mesas. The short-distance diffusion results in the shape evolution of film on each mesa and pit; while the long-distance diffusion results in the competitive growth of nanostructures on mesa and pit. In the following analysis, we assume that the short-distance diffusion is much faster than the long-distance diffusion. Hence, during the whole SSD process, films on both mesas and pits are always in their equilibrium shape corresponding to their instantaneous volume.

Our energetic analysis follows the following two steps: First, we obtain the equilibrium shape of film deposited on single mesa and single pit by comparing the interfacial energies of possible configurations. Next, using the results obtained for single mesa and single pit, we analyze the diffusion and evolution of film on a patterned substrate and obtain the phase diagrams of SSD.

#### Evolution of film deposited on a single mesa

**Figure 2:**
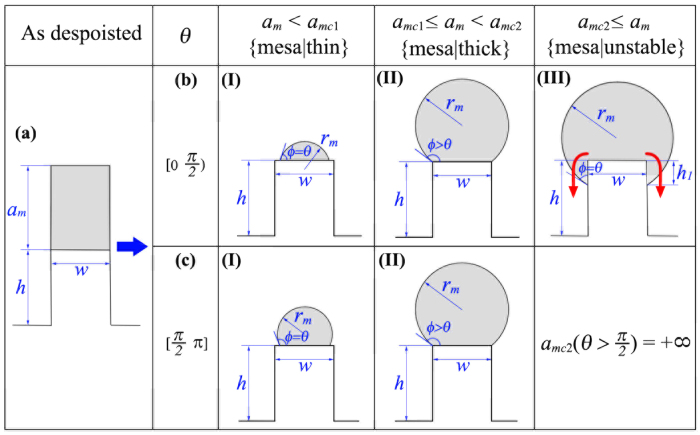
2D Morphological evolution of film deposited on a single mesa with width *w*, height *h* and film thickness *a*_*m*_. Depending on the wettability of film on the substrate (characterized as the wetting angle *θ*), the film will evolve to different equilibrium configurations. (**a**) initial configuration before annealing; (b-I), (b-II), (b-III) for decent wettability (*θ* < *π*/2) and (c-I), (c-II) for decreasing wettability (*θ* ≥ *π*/2). *r*_*m*_ is the radius of the resulted nanowire, *ϕ* is the contact angle between the film and the mesa, and *h*_1_ is the penetration depth. In configuration b-III, the film will slip down as indicated by the red arrows.

Figure 2 shows the result of 2D energetic analysis for the morphological evolution of film deposited on a single mesa. Before annealing, an initially uniform film with thickness *a*_*m*_ is deposited on the top of a mesa with width *w* and height *h* as shown in Fig. 1a. After deposition, temperature is elevated to accelerate the diffusion of film atoms. Depending on the thickness of film, three different regimes can be distinguished as shown in Fig. 2. (i) Regime I: For *a*_*m*_ < *a*_*m1*_(*θ*,*w*), only part of the mesa’s top is wetted by the film. The real contact angle is fixed to the value of *ϕ* = *θ* where *θ* is the wetting angle satisfying the Young relation. The wetting area in this case increases with increasing *a*_*m*_. Typical morphologies for regime I are given in Fig. 2b–I,2c–I for *θ* < *π*/2 and *θ* ≥ *π*/2, respectively; (ii) Regime II: For *a*_*m1*_(*θ*,*w*) ≤ *a*_*m*_ < *a*_*m2*_(*θ*,*w*), the film covers the mesa’s top completely. In this case, the wetting area is fixed and the wetting front is pinned by the ridges of the mesa. The contact angle *ϕ* is not fixed in this regime, it increases with increasing *a*_*m*_ and can have any value within the interval of *θ* ≤ *ϕ* ≤ *θ* + *π*/2. Typical morphologies for regime II are given in Fig. 2b-II,2c-II for *θ* < *π*/2 and *θ* ≥ *π*/2, respectively; and (iii) Regime III: For *a*_*m2*_(*θ*,*w*) ≤ *a*_*m*_, the contact front depins from the mesa’s ridges and climbs down from the top of mesa to the side walls. Our energetic analysis shows that, in this regime, the film on top of the mesa is unstable: the penetration depth *h*_1_ continues to increase until the whole film “slips down” from the mesa’s top surface (see the detailed discussion in Supplementary Information). Noticing that the depinning condition for regime III is equivalent to the condition of *ϕ* ≥ *θ* + *π*/2. In case of *θ* *>* *π*/2, this condition is unreachable since the value of *θ* + *π*/2 is larger than *π*, and thus Regime III does not exist in this case. For the convenience of subsequent analysis, we name the equilibrium configurations in Regime I, II and III as {mesa|thin}, {mesa|thick} and {mesa|unstable}, respectively.

It is noted that a similar energetic analysis for liquid on chemically patterned substrates was also performed^37^ and three regimes were identified. We find that Regimes 1 and 2 in ref. 37 are similar to Regimes I and II in our work. However, Regime 3 in ref. 37 is quite different from Regime III in our work: The liquid droplet in Regime 3 in ref. 37 is stabilized with the fixed contact angle, while the nanowire in Regime III in our current work is an unstable state. This unstable Regime III was also observed in previous KMC simulation^49^. Our results for film on single mesa also agree with those in ref. 48, which show that the maximum volume of a liquid droplet on individual pillars increases with increasing the wetting angle. The difference between Regime III in our work and Regime 3 in ref. 37 arises from the complexity of topologically patterned substrates. Compared with chemically patterned substrates, topologically patterned substrates require one more parameter, i.e., the penetration depth *h*_1_, to completely determine the geometry of film. This additional freedom results in the different evolution behaviors between Regime III here and Regime 3 in ref. 37.

Table 1 shows the detailed expressions for the variables listed in Fig. 1, including the critical thicknesses of *a*_*mc*1_ and *a*_*mc*2_, the radius of the film surface *r*_*m*_ and also the derivative of total interfacial energy *E*_*m*_ in case of single mesa with respect to the film thickness: *dE*_*m*_/*da*_*m*_.

#### Evolution of film deposited in a single pit

**Figure 3:**
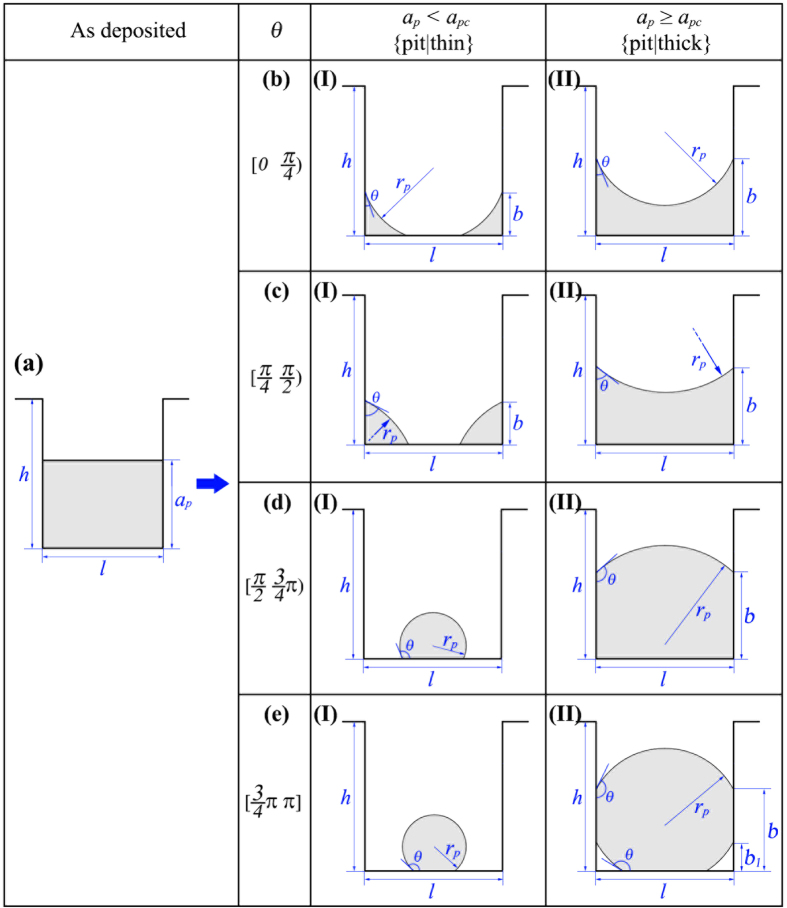
2D Morphological evolution of film deposited in a single pit with width *l*, height *h* and film thickness *a*_*p*_. Depending on the wettability of film on the substrate (characterized as the wetting angle *θ*), the film will evolve to different equilibrium configurations. (**a**) initial configuration before annealing; (b-I), (b-II) equilibrium configurations for *θ*∈[0, *π*/4; (c-I), (c-II) equilibrium configurations for *θ*∈[*π*/4,*π*/2); (d-I), (d-II) equilibrium configurations for *θ*∈[*π*/2, 3*π*/4); (e-I), (e-II) equilibrium configurations for *θ*∈[3*π*/4, *π*]. *r*_*p*_ is the radius of the film surface.

Figure 3a shows the initial configuration of a film deposited in a pit with height *h*, width *l* and film thickness *a*_*p*_. For simplification, we only study the deep pit case in this section, which means that the sidewall is assumed to be high enough to prohibit the film from spilling out of the pit. By enforcing this condition, our work distinguishes itself from that in ref. 46, which only dealt with small wetting angle and shallow pits. For small wetting angles and shallow pits (see the detailed discussion in Supplementary Information), our results are consistent with those in ref. 46. Hence, the results in ref. 46 can serve as an additional verification of our analysis.

When a solid film is deposited in a single pit, depending on the film thickness, two regimes can be distinguished as shown in Fig. 3. (i) Regime I: For *a*_*p*_ < *a*_*pc*_(*θ*,*l*), the film fails to connect the two sidewalls of the pit. Figure 3b–I,c–I,d–I,e–I show the typical equilibrium configurations for wetting angle in the range of *θ* < *π*/4, *π*/4 ≤ *θ* < *π*/2, *π*/2 ≤ *θ* < 3*π*/4 and 3*π*/4 ≤ *θ*, respectively. For *θ* < *π*/4, the initial continuous film splits into two corner wedges with a concave surface. This morphology is exactly the same as the cW phase in ref. 46. For *π*/4 ≤ *θ* < *π*/2, the film also splits into two corner wedges. But for this case, the film surface is convex. This morphology agrees with the cD phase in ref. 46. For *θ* ≥ *π*/2, the film detaches from the side walls and shrinks into a circular slice. (ii) Regime II: For *a*_*p*_ ≥ *a*_*pc*_(*θ*,*l*), the film is able to connect two sidewalls. Figure 3b-II,c-II,d-II,e-II show the typical equilibrium configurations for wetting angle in the range of *θ* < *π*/4, *π*/4 ≤ *θ* < *π*/2, *π*/2 ≤ *θ* < 3*π*/4 and 3*π*/4 ≤ *θ*, respectively. For *θ* < *π*/2, the film with a convex surface completely wets the bottom of the pit. For *π*/2 ≤ *θ* < 3*π*/4, the film also fully wets the bottom of the pit, but with a concave surface. For 3*π*/4 ≤ *θ*, the bottom of the pit is partially wetted and the surface of the film is concave. For the convenience of subsequent analysis, we name the equilibrium configurations in Regimes I and II as {pit|thin} and {pit|thick}, respectively. We notice, however, the missing of the morphology {pit|thick} in ref. 46, which can only appear in a relatively deep pit.

Table 2 lists the expressions of variables listed in Fig. 3, including the critical thickness *a*_*pc*_, the film’s surface radius *r*_*p*_ and the derivative of *E*_*p*_ with respect to the film thickness, *dE*_*p*_/*da*_*p*_, in terms of the film thickness *a*_*p*_, the wetting angle *θ* and the pit width *l*.

#### Evolution of film deposited on ripple-patterned substrates

After we obtained the equilibrium morphologies of film deposited on both single mesa and single pit, we can now study the morphological evolution of film deposited on patterned substrates by turning on the inter-diffusion between film on adjacent mesa and pit. During the SSD process, the total film volume keeps conservation, which indicates that:


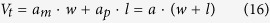


where, *a*_*m*_ and *a*_*p*_ are equivalent film thicknesses on mesa and pit, respectively, *V*_*t*_ is the volume of film per unit (one single mesa + one single pit). To study the inter-diffusion, we write the total interfacial energy of the patterned substrate as the sum of two parts belonging to film one mesa and film in pit, respectively, as following:


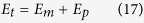


The actual diffusion direction is then determined by the derivative of the total interfacial energy with respect to the equivalent thickness *a*_*p*_:


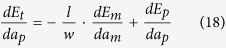


In case of *dE*_*t*_/*da*_*p*_> 0, the inter-diffusion is from pit to mesa, and in case of *dE*_*t*_/*da*_*p*_ < 0, the inter-diffusion is from mesa to pit. The relationship of *dE*_*t*_/*da*_*p*_ = 0 corresponds to boundaries in the phase diagram.

#### Phase diagram for small wetting angle: *θ* < *π*/2

**Figure 4:**
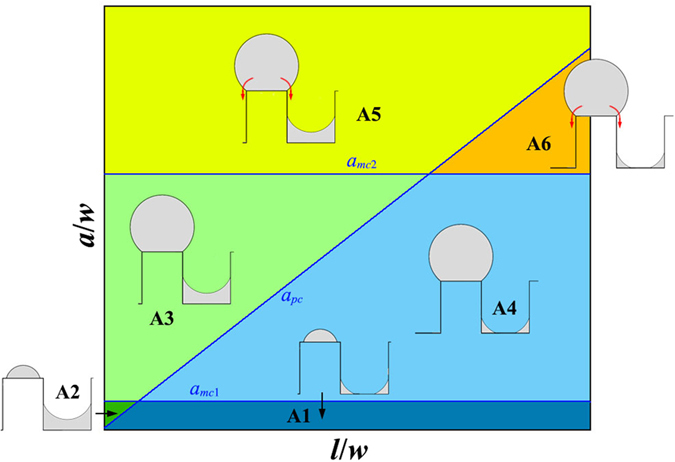
Subdivision of the (*l*/*w*, *a*/*w*) phase space for *θ* < π/2, each subspace corresponding to a different initial state from which the inter-diffusion begins. In case of small wetting angle, depending on *l*/*w* and *a*/*w*, there are six different subspaces: A1: {mesa|thin}-{pit|thin}, A2: {mesa|thin}-{pit|thick}, A3: {mesa|thick}-{pit|thick}, A4: {mesa|thick}-{pit|thin}, A5: {mesa|unstable}-{pit|thick} and A6: {mesa|unstable}-{pit|thin}.

Depending on the geometry of the substrate, the wetting angle and the film thickness, the inter-diffusion could begin from different initial states. Figure 4 shows the subdivision of the phase space of (*l*/*w*, *a*/*w*) in case of *θ* < *π*/2, each subspace corresponding to a different initial state which the inter-diffusion begins from. These initial states are achieved by combining the morphologies on mesa (as shown in Fig. 2) and morphologies in pits (as shown in Fig. 3). In case of *θ* < *π*/2, 6 different subspaces could be achieved as shown in Fig. 4. We use rule of {∙}-{∙} to name these initial states, the first part before the “-” is the initial morphology on mesa, and the second part after the “-” is the initial morphology in pit. In the following, we analyze the direction of the inter-diffusion based on Equation 18, and study the morphological evolution of film starting from each initial states separately.

##### Subspace A1

In subspace A1, the film thickness is smaller than both *a*_*mc*1_ and *a*_*pc*_, so the initial state before annealing is {mesa|thin}-{pit|thin} (see Fig. 4). According to Equations 2, in case of *θ* < *π*/4, we have:


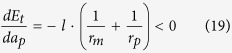


This means that in subspace A1, if the wetting angle is smaller than *π*/4, the inter-diffusion will be always from mesa to pit. As a result, the final equilibrium morphology will be {pit|thin} or {pit|thick}, depending on the film thickness.

And in case of *θ* ≥ *π*/4, substituting Equation 2 and Equation 11 into Equation 18, we have:


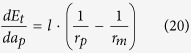


In this case, the inter-diffusion can be either from mesa to pit or reversed, depending on the relative value of *r*_*p*_ and *r*_*m*_. Based on Equation 3 and Equation 12, the relationship of *r*_*p*_ = *r*_*m*_ gives a critical pit width *l*_*A*1_ as:


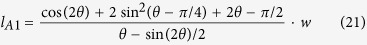


In case of *l* < *l*_*A*1_, the inter-diffusion is from pit to mesa, the equilibrium morphology corresponding to this case is {mesa|thin}. However, in case of *l* ≥ *l*_*A*1_, the inter-diffusion is from mesa to pit, and the equilibrium morphology corresponding to this case can be either {pit|thin} or {pit|thick}, depending on the film thickness.

##### Subspace A2

In subspace A2, the film thickness is within the range of [*a*_*pc*_ *a*_*mc*1_], so the initial state before annealing is {mesa|thin}-{pit|thick} (see Fig. 4). Substituting Equation 2 and Equation 8 into Equation 18, we obtain the following result for both *θ* < *π*/4 and *θ* ≥ *π*/4:


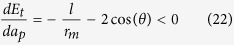


This means that in subspace A2, the inter-diffusion is always from mesa to pit, the equilibrium morphology corresponding to subspace A2 is {pit|thick}.

##### Subspace A3

In subspace A3, the film thickness is within the range of [max(*a*_*pc*_ *a*_*mc*1_), *a*_*mc*2_], the initial state before annealing is {mesa|thick}-{pit|thick} (see Fig. 4). Substituting Equation 4 and Equation 8 into Equation 18, we obtain the following result for both *θ* < *π*/4 and *θ* ≥ *π*/4:


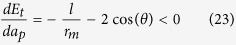


This means that in subspace A3, the inter-diffusion is always from mesa to pit, the equilibrium morphology corresponding to subspace A3 is {pit|thick}.

##### Subspace A4

In subspace A4, the film thickness is within the range of [*a*_*mc*1_, min(*a*_*pc*_,*a*_*mc*2_)], the initial state before annealing is {mesa|thick}-{pit|thin} (see Fig. 4). In case of *θ* < *π*/4, substituting Equation 4 and Equation 10 into Equation 18, we have:


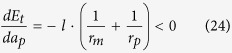


The inter-diffusion is from mesa to pit, the equilibrium morphology corresponding to this case can either be {pit|thin} or {pit|thick}, depending on the film thickness.

In case of *θ* ≥ *π*/4, substituting Equation 4 and Equation 11 into Equation 18, we have:


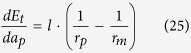


In this case, the direction of inter-diffusion is determined by the relative value of *r*_*p*_ and *r*_*m*_. However, after further calculation based on Equation 5 and Equation 12, we prove that in subspace A4, the radius of *r*_*m*_ is always smaller than *r*_*p*_, which implies that the inter-diffusion in subspace A4 is always from mesa to pit, and the equilibrium morphology corresponding to subspace A4 could be either {pit|thin} or {pit|thick} depending on the film thickness.

##### Subspace A5

In subspace A5, the film thickness is larger than both *a*_*mc*2_ and *a*_*pc*_, the initial state before annealing is {mesa|unstable}-{pit|thick} (see Fig. 4). In this subspace, the film on mesa is unstable and will slip down to pit, the corresponding equilibrium morphology is {pit|thick}.

##### Subspace A6

In subspace A6, the film thickness is in region of [*a*_*mc*2_ *a*_*pc*_], the initial state before annealing is {mesa|unstable}-{pit|thin} (see Fig. 4). In this substrate, the film deposited on mesa is unstable and will slip down to pit, the corresponding equilibrium morphology can be either {pit|thin} or {pit|thick} depending on the film thickness.

#### Equilibrium morphology in case of inter-diffusion from mesa to pit

As shown above, in subspaces A1, A4 and A6, the inter-diffusion is from mesa to pit and the corresponding equilibrium morphology can be either {pit|thin} or {pit|thick} depending on the quantity of film deposited. In these three subspaces, initially the film in pit separates itself into two corner wedges with each adsorbed to one of the side walls, i.e. {pit|thin}. During the inter-diffusion, these two corner wedges grow continuously, and if the amount of film atoms diffused into pit is enough, these two growing wedges will meet with each other and then transform into the morphology of {pit|thick}. The critical film thickness *a*_*A*1_ for this transformation is given by:


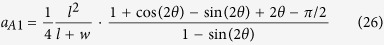


In subspaces A1, A4 and A6, the corresponding equilibrium morphology is {pit|thin} in case of *a* ≤ *a*_*A*1_, and {pit|thick} in case of *a > a*_*A*1_.

#### Phase diagram for large wetting angle: *θ* ≥ *π*/2

**Figure 5:**
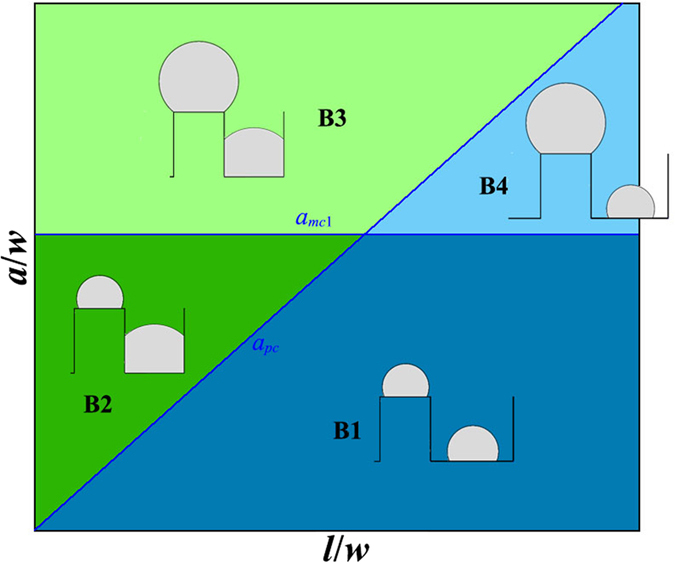
Subdivision of the (*l*/*w*, *a*/*w*) phase space for *θ* ≥ *π*/2, each subspace corresponding to a different initial state from which the inter-diffusion begins. In case of large wetting angle, depending on *l*/*w* and *a*/*w*, four different subspaces can be distinguished: B1: {mesa|thin}-{pit|thin}, B2: {mesa|thin}-{pit|thick}, B3: {mesa|thick}-{pit|thick} and B4: {mesa|thick}-{pit|thin}.

Figure 5 shows the subdivision of the (*l*/*w*, *a*/*w*) phase space in case of *θ* ≥ *π*/2, each subspace corresponds to a different initial state for beginning of the inter-diffusion. In case of *θ* ≥ *π*/2, 4 different subspaces could be achieved as shown in Fig. 5. In the following, we analyze the inter-diffusion direction and the morphological evolution for each subspace.

##### Subspace B1

In subspace B1, the film thickness *a* is smaller than both *a*_*mc*1_ and *a*_*pc*_, the initial state before annealing is: {mesa|thin}-{pit|thin} (see Fig. 5). Substituting Equation 2 and Equation 13 into Equation 18, we obtain the following result for both *π*/2 ≤ *θ* < 3*π*/4 and 3*π*/4 ≤ *θ* ≤ *π*:


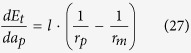


In subspace B1, the direction of the inter-diffusion depends on the relative value of *r*_*m*_ and *r*_*p*_. If the *r*_*m*_ is larger than *r*_*p*_, the inter-diffusion will be from pit to mesa. However, if *r*_*m*_ is smaller than *r*_*p*_, the inter-diffusion will be from mesa to pit. The phase boundary is determined by the relationship of *r*_*m*_ = *r*_*p*_. Based on Equation 3 and Equation 14, *r*_*m*_ = *r*_*p*_ is equivalent to:


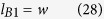


where, *l*_*B*1_ is a critical pit width. In case of *l* < *l*_*B*1_, the inter-diffusion is from pit to mesa, the corresponding equilibrium morphology in this case can be either {mesa|thin} or {mesa|thick} depending on the film thickness. And in case of *l* ≥ *l*_*B*1_, the inter-diffusion is from mesa to pit, the corresponding equilibrium morphology in this case can be either {pit|thin} or {pit|thick} depending on the film thickness.

##### Subspace B2

In subspace B2, the film thickness *a* is in the range of [*a*_*pc*_, *a*_*mc*1_], the initial state before annealing is {mesa|thin}-{pit|thick} (see Fig. 5). Substituting Equation 2 and Equation 8 into Equation 18, we obtain:


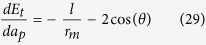


The inter-diffusion in subspace B2 depends on the radius *r*_*m*_, or equivalently, the film thickness. If the film thickness is smaller than a critical value of *a*_*B*2_, the inter-diffusion is from mesa to pit and the equilibrium morphology corresponding to this case is {pit|thick}. However, if *a* ≥ *a*_*B*2_, the inter-diffusion will be from pit to mesa and the equilibrium morphology corresponding to this case can be either {mesa|thin} or {mesa|thick} depending on the film thickness. Based on Equation 3 and Equation 29, the critical thickness of *a*_*B*2_ can be expressed by:


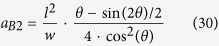


##### Subspace B3

In subspace B3, the film thickness is larger than both *a*_*pc*_ and *a*_*mc*1_, the initial state before annealing is {mesa|thick}-{pit|thick} (see Fig. 5). Substituting Equation 4 and Equation 8 into Equation 18, we obtain:


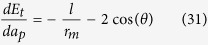


Same as in subspace B2, the inter-diffusion can also be divided into two cases, depending on the radius of *r*_*m*_ or the film thickness. A critical thickness of *a*_*B*3_ can be obtained for subspace B3:


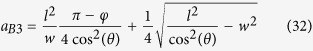


where


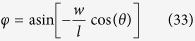


In case of *a* < *a*_*B*3_, the inter-diffusion is from mesa to pit, the equilibrium morphology corresponding to this case is {pit|thick}. And in case of *a* ≥ *a*_*B*3_, the inter-diffusion is from pit to mesa, {mesa|thick} is the equilibrium morphology corresponding to this case.

##### Subspace B4

In subspace B4, the film thickness is in the range of [*a*_*mc*1_, *a*_*pc*_], the initial state before annealing is {mesa|thick}-{pit|thin} (see Fig. 5). Substituting Equation 4 and Equation 13 into Equation 18, we obtain:


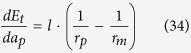


The direction of inter-diffusion depends on the relative value of *r*_*p*_ and *r*_*m*_. Based on Equation 5 and Equation 14, we prove that in subspace B4, the radius of *r*_*p*_ is always larger than *r*_*m*_, which implies that the inter-diffusion in subspace B4 is always from mesa to pit. The final equilibrium morphology can either be {pit|thin} or {pit|thick} depending on the film thickness.

#### Equilibrium morphology in case of inter-diffusion from pit to mesa

As described above, in subspaces B1 and B2, an additional criterion should be given to distinguish the equilibrium morphology between {mesa|thin} and {mesa|thick} in case of inter-diffusion from pit to mesa. This additional criterion is given in the following:


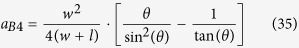


For *a* < *a*_*B*4_, the equilibrium morphology is {mesa|thin}. However, for *a* ≥ *a*_*B*4_, the equilibrium morphology is {mesa|thick}.

#### Equilibrium morphology in case of inter-diffusion from mesa to pit

As described above, an additional criterion is required in subspaces B1 and B4 to distinguish the equilibrium morphologies of {pit|thin} and {pit|thick} in case of inter-diffusion from mesa to pit. In these two subspaces, initially the film deposited in pit forms a single nanowire. During the inter-diffusion, the nanowire grows continuously. Once the radius of the nanowire *r*_*p*_ exceeds *l*/2, it will contact with side walls and transform into the morphology of {pit|thick}. The criterion for this transformation is given as following:


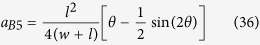


In case of *a* < *a*_*B*5_, the equilibrium morphology is {pit|thin}. And in case of *a* ≥ *a*_*B*5_, the equilibrium morphology is {pit|thick}.

#### Cassie-Baxter state in case of inter-diffusion from pit to mesa

In case of inter-diffusion from pit to mesa, a special case should be considered separately, in which the radius of the nanowires grown on mesas becomes so large that they start to contact with each other. In this case, the nanowires on mesas will merge with each other and form a flat film standing on all mesas to minimize the interfacial energy. This state is a pure Cassie-Baxter state^50^ (CB1 state). The critical film thickness of the CB1 state is given by:


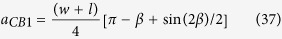


where


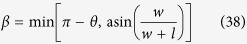


It should be noticed that besides the CB1 state, there may also exist another CB2 state, in which the film deposited on mesa is so thick that the nanowires on mesas trend to contact with each other even at the initial state before inter-diffusion. The critical film thickness for this CB2 state is given by:


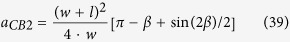


In our analysis, we do not consider any defects such as the surface roughness of the film, variation of the initial film thickness and presence of grain boundaries, so pure CB state can be achieved. However, in real cases, the existence of these defects may result in the formation of a partial CB state near the boundary given by Equation 37. In a partial CB state, large particles standing on multi-mesas will form^51^.

#### Spilling and Wenzel state in case of inter-diffusion from mesa to pit

In our analysis, we assume that the pit is deep enough to hold all film material. However, in case of finite pit depth, more consideration should be included. Usually, as the pit becomes shallow, the three-phase contact line will be pinned by ridges of mesas, further decrease of the depth will then result in the over spilling of film from pits and in some case result in the formation of purely Wenzel state^52^, where the whole patterned substrate is fully wetted by the film. We put the detailed discussion of the influence of pit’s depth in the Supplementary Information.

#### Phase diagrams and comparing with experimental results

##### Phase diagrams

**Figure 6:**
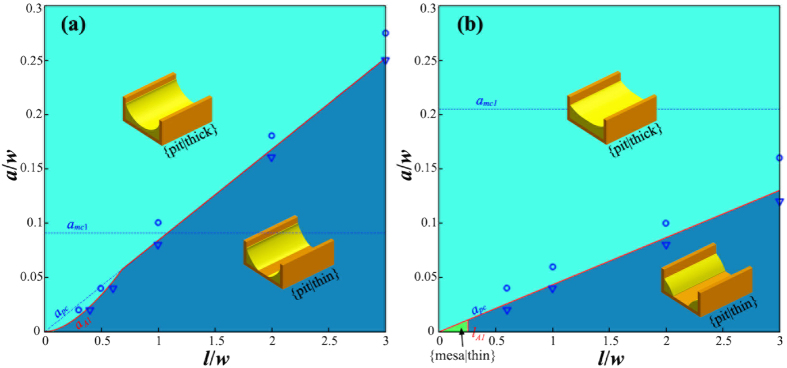
Phase diagram for a wetting angle of (**a**) *θ* = *π*/6 and (b) *θ* = *π*/3. Regions with different colors represent different equilibrium morphologies. The insets illustrate the phase field simulation results. Solid red lines: phase boundary predicted by the energetic analysis; dashed blue lines: critical thicknesses of *a*_*mc*1_ and *a*_*pc*_; and blue hollow circular and triangular dots: the phase field simulation results agree with the morphologies of {pit|thick} and {pit|thin}, respectively.

Based on the above energetic analysis, we plot the phase diagrams for patterned SSD with a given wetting angle. Figure 6a,b show the phase diagram for *θ* = *π*/6 and *θ* = *π*/3, respectively. In Fig. 6, the solid red line is the boundaries to distinguish different equilibrium morphologies (or phases), and the dashed blue line shows the initial subdivision of the phase space same as in Fig. 4. We can see that for a wetting angle of *θ* < *π*/4, the film material can only be stable in the pits, i.e., only {pit|thin} and {pit|thick} are the stable morphology. For a wetting angle larger than *π*/4, besides {pit|thin} and {pit|thick}, there also exists a very narrow region in which {mesa|thin} is the minimal free energy morphology. It should be noted that, in the present paper, we assume that only one single nanowire will form on each mesa. This requires that the value of *a*/*w* should not too small so as to avoid the edge pinch-off effect^53^. According to ref. 53, the critical ratio of *a*/*w* is about 0.006 for *θ* = *π*/3, which is within the upper-middle of the region of {mesa|thin}. Therefore, in reality, the phase of {mesa|thin} in Fig. 6b may not exist because of the edge pinch-off.

Besides energetic analysis, we also performed phase field simulation to further verify our results. (See Supplementary Information for the details of the phase field model). We show in Fig. 6 the typical phase field simulation results for each phase. The blue hollow circular and triangular dots in Fig. 6 represent the phase field simulation results which agree with the equilibrium morphologies of {pit|thick} and {pit|thin}, respectively. And the small insets in Fig. 6 show the typical simulated morphologies according to the corresponding phase region. It can be seen that the phase boundary predicted by our energetic analysis agrees very well with the phase field simulation.

**Figure 7:**
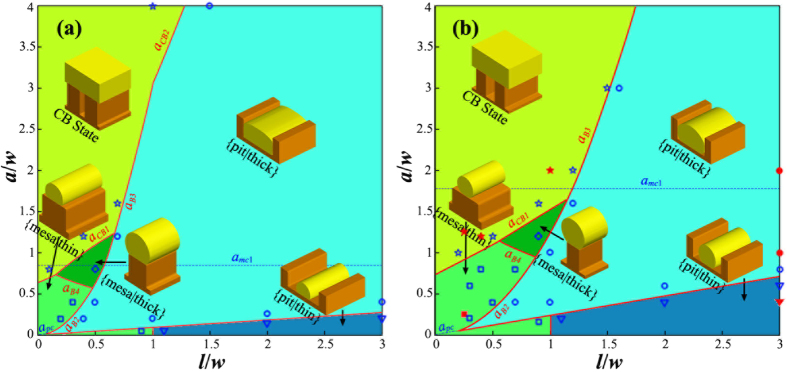
Phase diagram for a wetting angle of (**a**) *θ* = 120° and (**b**) *θ* = 140°. Regions with different colors represent different equilibrium morphologies. The insets illustrate phase field results in their corresponding regions. Solid red lines: phase boundary predicted by the energetic analysis; dash blue lines: critical thicknesses of *a*_*mc*1_ and *a*_*pc*_; blue hollow circular, triangular, square, diamond and pentagram dots: phase field simulation results agree with the morphologies of {pit|thick}, {pit|thin}, {mesa|thin}, {mesa|thick} and CB state, respectively; and red solid circular, triangular, square and pentagram dots: experimental results agree with the morphologies of {pit|thick}, {pit|thin}, {mesa|thin} and CB state, respectively.

Figure 7a,b show the phase diagrams for *θ* = 120° and *θ* = 140°, respectively. In Fig. 7, the solid red line is the boundaries to distinguish different equilibrium morphologies (or phases), and the dashed blue line shows the initial subdivision of the phase space same as in Fig. 5. Comparing Fig. 7 to Fig. 6, we can see that the morphological evolution of SSD in case of *θ* ≥ *π*/2 is much more complex than the case of *θ* < *π*/2: there are 5 different equilibrium phases for *θ* ≥ *π*/2, while there are only 2 for *θ* < *π*/2. In case of *θ* ≥ *π*/2, as the wetting angle increases, the phase space occupied by morphologies of {mesa|thin}, {mesa|thick} and CB state expands, however, the phase space occupied by rest equilibrium morphologies which are stable in pits shrinks. In case of *θ* = 120°, there are two CB state boundaries, that is, CB1 and CB2, but for *θ* = 140°, there is only CB1 boundary but no CB2 boundary.

Besides the phase boundaries predicted by our energetic analysis, Fig. 7 also shows typical phase field simulation results for each phase. The blue hollow square, diamond, pentagram, circular and triangular dots representing the phase field simulation results agree with the morphologies of {mesa|thin}, {mesa|thick}, CB state, {pit|thick} and {pit|thin}, respectively. We can see that an excellent agreement has been achieved between the phase field simulations and the energetic analysis.

From phase diagrams in Figs 6 and 7, we conclude that the dewetting behavior in small wetting angle condition is relatively simple: the film can only be stable in pits, this is consistent with many of previous studies for liquid layers on substrates with either chemically^31^,^32^,^33^,^34^,^35^,^36^,^37^,^38^,^39^,^40^,^41^,^42^ or topologically^41^,^42^,^43^,^44^,^45^,^46^,^47^,^48^ patterned. However, for large wetting angle, the dewetting behavior is much more complex even in the simple deep pit condition as considered in current paper: the film can be either stable in pits or on mesas, which gives us an alternative method to control the position and arrangement of the desired nanostructure by operating on the substrate geometry and film thickness in addition to on the wetting angle as in cases of chemically patterned substrate.

##### Comparing with experimental results

In addition to the phase field simulations, we also performed dewetting experiments to verify our energetic analysis. We fabricated pseudo-3D structures consisting of 10 microns long mesas with a height of 100 nm, and well-defined widths and periodicities. These mesa structures were formed on silicon substrates with native oxide intact using electron beam lithography (EBL) crosslinking of hydrogen silsesquioxane (HSQ), a high-contrast negative-tone resist^54^,^55^. Cross-linked HSQ resembles amorphous SiO_x_. Since the roughness of the silicon substrates prepared by the above method is typically below 1 nm, which is much smaller than the geometrical size, its influence can be reasonably considered by choosing a proper wetting angle. Gold was deposited on the template via electron-beam evaporation technique at room temperature. The metal dewetting was induced via thermal annealing at 400 °C (the melting point for Au nanoparticles with a radius larger than 10 nm is about 1000 °C) in a rapid thermal processor for 5–30 minutes^14^. The wetting angle between Au and Si with oxide layer is about 140° 56. The red solid square, pentagram, circular and triangular dots in Fig. 7b representing the experimental results agree with the morphologies of {mesa|thin}, CB state, {pit|thick} and {pit|thin}, respectively. The experimental results agree well with the energetic analysis. In the following, we present these experimental results together with our phase field simulations.

##### {mesa|thin} phase of (*l*/*w* = 0.25, *a*/*w* = 0.25)

**Figure 8:**
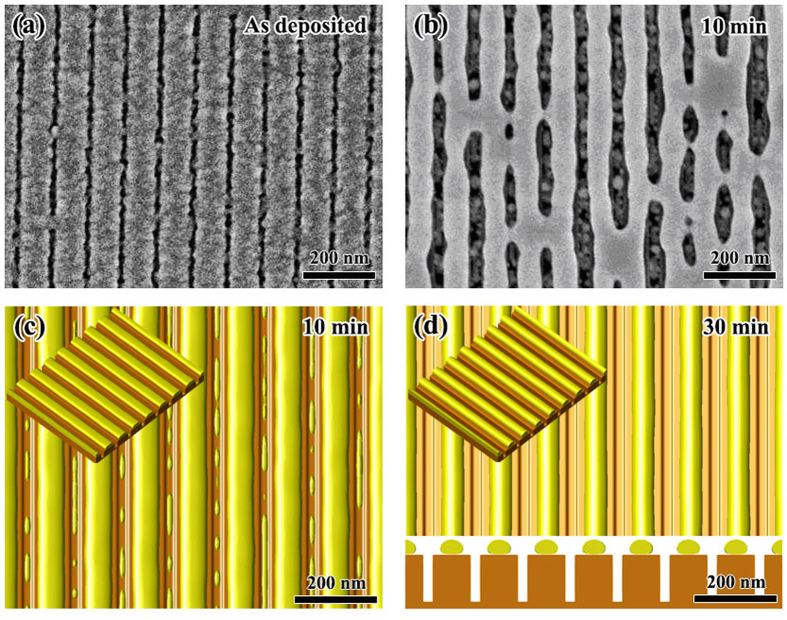
Morphological evolution of *w* = 80 nm, *l* = 20 nm and *a* = 20 nm. (**a**,**b**) Are the experimental observation before and after annealing at 400 °C for 10 min, respectively; (**c,d**) are the top view together with 3D view (small figure at the upper-left corner) of the phase field simulation results for 10 min and 30 min, respectively. (**d**) Also shows the cross-section of the film together with substrate for 30 min.

According to Fig. 7b, the point of (*l*/*w* = 0.25, *a*/*w* = 0.25) is located in the region of {mesa|thin} phase. In our experiment, we chose following geometrical parameters for this case: *w* = 80 nm, *l* = 20 nm and *a* = 20 nm. Figure 7 shows the experimental observation together with our phase field simulation result for this sample. We can see that after 10 min annealing at 400 °C, the Au film on mesas slightly shrinks and partially covers the mesa top. We can also see that the Au film in pits becomes discontinuous and breaks up into many small particles after annealing. This experimental observation agrees well with our phase field simulation as shown in Fig. 8c. Our phase field simulation result shows that for this sample, the Au atoms diffuse from pit to mesa, and evolve into a {mesa|thin} morphology after about 15 min. Figure 8d shows the simulated morphology after 30 min annealing. It should be noted here that, because of the sputtering nature, the Au film deposited in our experiments is in a polycrystalline structure. The existence of grain boundaries (GBs) and their evolution also have influence on the SSD process. Our study shows that the GBs will make the SSD morphologies irregular, resulting in the formation of undesired defects such as the crosslinks between adjacent nanowires as shown in Fig. 8b. Thus, in order to eliminate the influence of GB and to produce perfect morphologies as predicted by the energetic analysis, single-crystalline film is optimal. Another consideration for polycrystalline films is that the annealing time should be carefully controlled to minimize the coarsening between grains, which may also destroy the desired morphologies.

##### CB state of (*l*/*w* = 0.25, *a*/*w* = 1.25)

**Figure 9:**
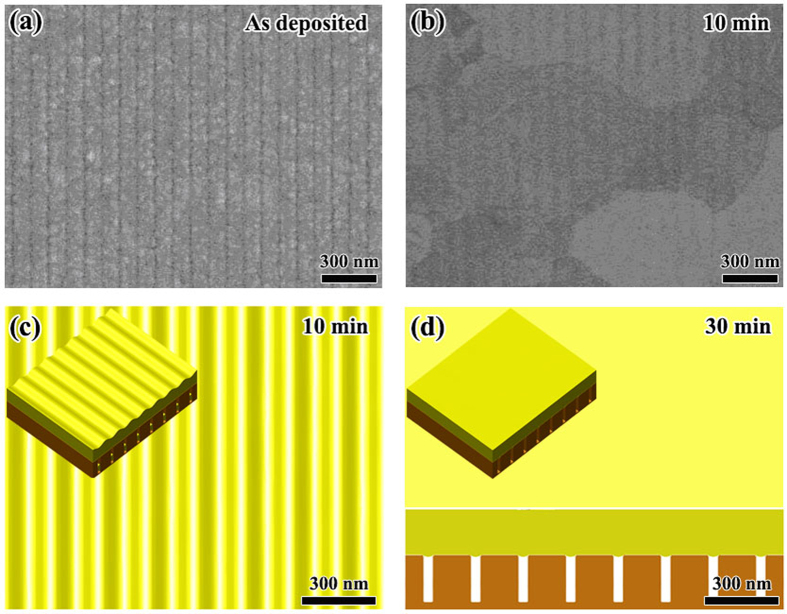
Morphological evolution for w = 80 nm, l = 20 nm and a = 100 nm. (**a,b**) Respectively show the experimental observation before and after annealing at 400 °C for 10 min. (**c,d**) show the top view together with 3D view (inset) of the phase field simulation results for 10 min and 30 min, respectively. (**d**) Also shows the cross-section for 30 min.

According to Fig. 7b, the point of (*l*/*w* = 0.25, *a*/*w* = 1.25) is located in the region of CB state. We chose the following geometrical parameters for this case: *w* = 80 nm, *l* = 20 nm and *a* = 100 nm. Figure 9 shows the experimental observations together with the phase field simulation results. We can see that after 10 min annealing at 400 °C, the Au film becomes nearly flat and no pits can be seen in Fig. 9b. At 10 min, the grains in the Au film grow dramatically compared to the as deposited state, grain size increases from several nanometers before annealing to several hundreds of nanometers after annealing which is much bigger than the period of the ripple-pattern, this observation implies that the Au film is freely standing on the mesas and there are no obstacles for the migration of GBs. In Fig. 9b, a light contrast oscillation also coexists with the GBs. Figure 9c,d show the phase field simulation results for this sample. It is seen that after 10 min annealing, a continue film standing on all mesas already is reach (CB state). However, at 10 min, the surface of the film is still unflatten, small perturbations with same period as the substrate exist as shown in Fig. 9c, this result agrees with the light contrast oscillation observed in Fig. 9b. At 10 min, there is still remaining Au in pits. With further annealing, the surface is becoming more and more flat, and eventually becomes completely flat at 30 min as shown in Fig. 9d. Besides the point of (*l*/*w* = 0.25, *a*/*w* = 1.25), our experiments also show that samples of (*l*/*w* = 0.4, *a*/*w* = 1.2) and (*l*/*w* = 1, *a*/*w* = 2) also evolve into CB state as shown in Fig. 7b. The geometrical parameters for these two cases are: *w* = 50 nm, *l* = 20 nm, *a* = 60 nm and *w* = 50 nm, *l* = 50 nm, *a* = 100 nm, respectively. The CB state which allows air-filled gaps to form under a continuous metal film will be useful in fabricating air-filled electronic devices with parasitic capacitances^57^.

##### {pit|thin} phase of (*l*/*w* = 3, *a*/*w* = 0.4)

**Figure 10:**
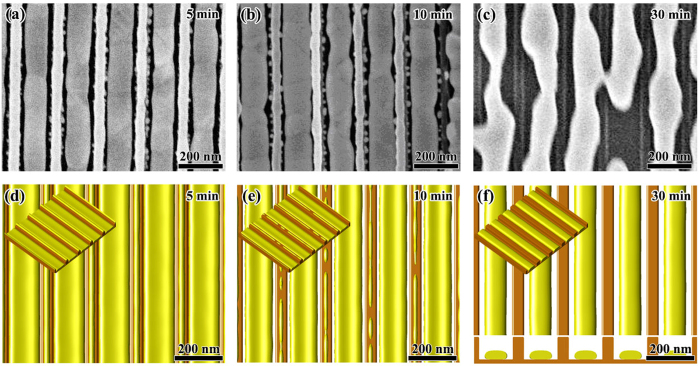
Morphological evolution of *w* = 50 nm, *l* = 150 nm and *a* = 20 nm. (**a–c**) Show respectively the experimental observation for annealing after 5, 10 and 30 min. (**d–f**) Show the top view together with 3D view (inset) of the phase field simulation for 5, 10 and 30 min, respectively. (**f**) Also shows the cross-section for 30 min.

According to Fig. 7b, the point of (*l*/*w* = 3, *a*/*w* = 0.4) is located in the region with the {pit|thin} configuration. We chose the following geometrical parameters for this case: *w* = 50 nm, *l* = 150 nm and *a* = 20 nm. Figure 10 shows the experimental observations together with the phase field simulation results. We can see that the phase field simulation agrees with the experimental observation. After annealing for 5 min, the Au film in pits shrinks and partially covers the pits. After 10 min annealing, some of the mesa tops are devoid of Au, and the Au material originally on these tops diffuses into adjacent pits. After annealing for 30 min, almost all the Au material on mesa tops diffuses down to pits and form a {pit|thin} configuration. Comparing with Fig. 10c,f, we see that the morphology observed in experiment is not very ideal as shown in the phase field simulation. In Fig. 10c, some parts of the Au film in pits become discontinuous and there also exist several crosslinks between adjacent pits. These imperfections arise from the influence of grain boundaries.

##### {pit|thick} phase of *(l*/*w* = 3, *a*/*w* = 1)

**Figure 11:**
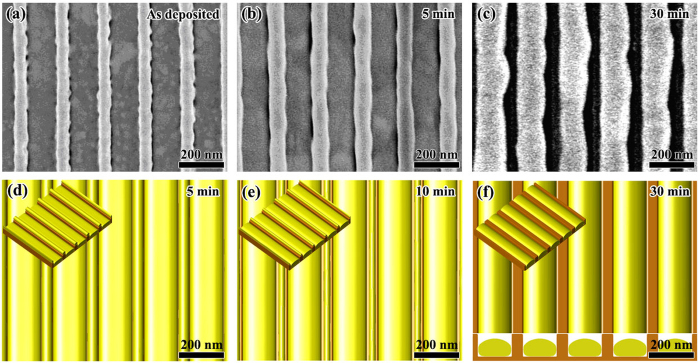
Morphological evolution of *w* = 50 nm, *l* = 150 nm and *a* = 50 nm. (**a–c**) Show respectively the experimental observation before annealing and after annealing at 400 °C for 5 min and 30 min. (**d–f**) Show the top view together with 3D view (inset) of the phase field simulation for 5 min, 15 min and 30 min, respectively. (**f**) Also shows the cross-section for 30 min.

According to Fig. 7b, the point of (*l*/*w* = 3, *a*/*w* = 1) is located in the region of {pit|thick} phase. The geometrical parameters for this case are: *w* = 50 nm, *l* = 150 nm and *a* = 50 nm. Figure 11 shows the experimental observations together with the phase field simulation results. We can see that after annealing at 400 °C for 5 min, no obvious shrinkage of the Au film in pits can be observed as compared with the case of (*l*/*w* = 3, *a*/*w* = 0.4) (see Fig. 10a,d). Our phase field simulation also shows that for this sample, at 5 min annealing, there is no obvious change from the top view, which agrees well with the experimental observation (Fig. 11b). After further annealing, the Au atoms diffuse from mesas to pits and finally form a {pit|thick} morphology as shown in Fig. 11f at 30 min. This result also agrees well with the experimental observation shown in Fig. 11c, in which all the Au on mesas has diffused into pits.

##### Wenzel state of (*l*/*w* = 3, *a*/*w* = 2)

**Figure 12:**
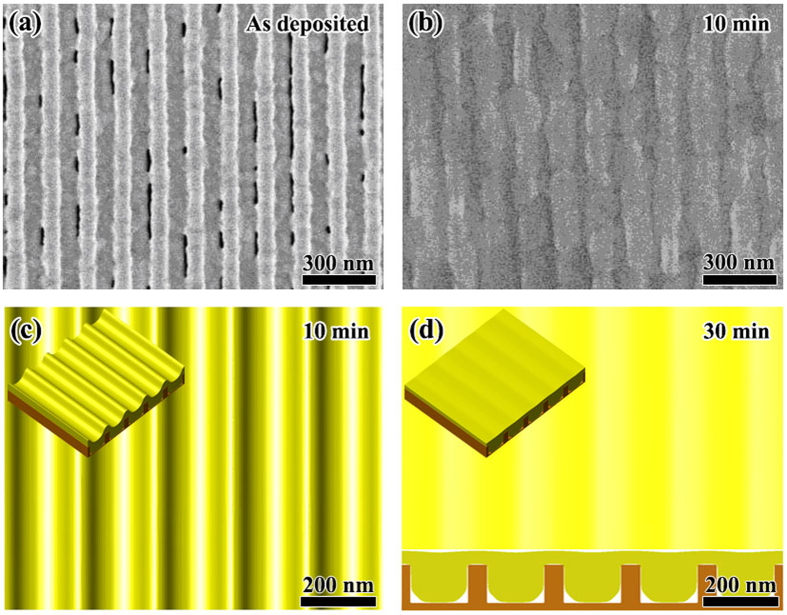
Morphological evolution of *w* = 50 nm, *l* = 150 nm, *a* = 100 nm and *h* = 100 nm. (**a**,**b**) Respectively show the experimental observation before and after annealing at 400 °C for 10 min. (**c,d**) Show the top view together with 3D view (inset) of the phase field simulation of 10 min and 60 min, respectively. (**d**) Also shows the cross-section for 60 min.

We have pointed out that, in case of inter-diffusion from mesas to pits, if pits are not deep enough, the film will spill out from pits and form WZ state. This is the case of the following parameters in experiments: *w* = 50 nm, *l* = 150 nm, *a* = 100 nm and *h* = 100 nm. Figure 12 shows the experimental observations together with the phase field simulation results. We can see that after annealing at 400 °C for 10 min, the Au film completely covers the whole Si substrate, and no mesas can be seen from the top view. Different from the case of CB state as shown in Fig. 9b, the grain size for this sample is still small and shows an elongated shape along the ripple direction. This result implies that the GBs in this sample cannot migrate freely. Figure 12c,d show the phase field simulation results for this sample after 10 min and 60 min annealing, respectively. We can see that for this sample, the Au film forms a WZ state. At 10 min, the surface of the film is not flat. The surface roughness slowly deceases during further annealing and becomes near flat at about 60 min as shown in Fig. 12d.

## Conclusion

We have studied the solid state dewetting process on ripple-patterned substrates by performing 2D energetic analysis, phase field simulations and experiments. Our results show that the morphological evolution of patterned SSD is sensitive to the wetting angle, film thickness and substrate geometry. As a result, a rich variety of different stable morphologies can be achieved by controlling of these parameters. Based on energetic analysis, we presented phase diagrams for SSD on ripple-patterned substrates. The predicted phase diagrams agree well with both phase field simulations and experimental results for the Au/Si film/substrate system, thus verifying our theoretic analysis. Hence, the phase diagrams presented here provide useful guidelines for using solid-state dewetting as a tool to achieve various nanostructures on patterned substrates. Here, we should notice that our experiments show that defects such as grain boundaries tend to destroy the well-ordered morphologies predicted in our energetic analysis and phase field simulations by forming breaks and crosslinks. To minimize such degeneration, one should carefully control the annealing time, or use single-crystal film instead.

## Methods

### Simulations

A three-dimensional phase field simulation model was developed. Briefly, the three-dimensional evolution of the phase fields was obtained by solving the Cahn-Hilliard equation using the Fourier-Spectral method. The simulations were performed on a workstation using a custom algorithm written in MATLAB (Mathworks, Massachusetts, USA). The details of the model and the parameters used are provided in the Supplementary Information.

### Electron beam lithography

HSQ (Product number: XR-1541-006 (6%), Dow Corning, Michigan, USA) was spun coated onto a silicon substrate to thicknesses of 100 nm. Electron beam lithography (EBL) was performed using an Elionix ELS-7000 system with an accelerating voltage of 100 kV and a beam current of 500 pA. The exposure field size was 75 μm × 75 μm with a step size of 0.3125 nm. Room temperature development of exposed HSQ is done in a salty developer (1% NaOH + 4% NaCl wt/wt in DI water) for 1 minute. The sample was immersed in DI water for 1 minute to stop the development, after which the sample was immersed in IPA before blow-drying with a steady stream of nitrogen gas.

### Metal deposition

Gold was deposited with an electron-beam evaporator (Explorer Coating System, Denton Vacuum, New Jersey, USA) at 0.5 Å/s. The chamber pressure during the process was ~5 × 10^−7^ torr. The sample holder was not rotated during the evaporation process. No adhesion layer was used.

### Thermally induced dewetting

The sample was heated up to induce dewetting. To do so, a rapid thermal processing system (Jetfirst, Jipelec, Grenoble, France) was used with the following schedule: 120 sec ramp up from room temperature to 400 °C, 5 to 30 minutes at 400 °C, and 30 s ramp down to room temperature.

### Scanning electron microscopy characterization

The samples were characterized using scanning electron microscopy (Elionix Inc., Tokyo, Japan) at 5 or 10 kV with a working distance of 5 mm.

## Additional Information

**How to cite this article**: Lu, L.-X. *et al*. Nanostructure Formation by controlled dewetting on patterned substrates: A combined theoretical, modeling and experimental study. *Sci. Rep*. **6**, 32398; doi: 10.1038/srep32398 (2016).

## Supplementary Material

Supplementary Information

## Figures and Tables

**Table 1:** Expressions for the derivative of the total energy with respect to the film thickness, radius *r*_*m*_ and the critical film thicknesses for the evolution of film deposited on a single mesa.

*a*_*m*_	Configurations	*dE*_*m*_/*da*_*m*_	*r*_*m*_
[0, *a*_*mc*1_)	2b-I, 2c-I	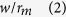	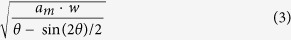
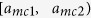	2b-II, 2c-II	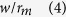	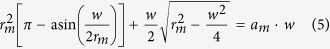
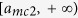	2b-III	Unstable	
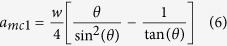		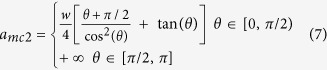	

**Table 2:** Expressions for the derivative of the total energy with respect to the film thickness, the radius *r*_*p*_, and the critical film thickness for the evolution of the film deposited in a single pit.

*a*_*p*_	Configurations	*dE*_*p*_/*da*_*p*_	*r*_*p*_
[0, *a*_*pc*_)	3b-II, 3c-II,3d-II, 3e-II	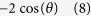	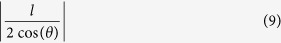
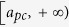	3b-I	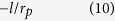	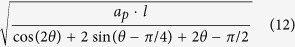
	3c-I	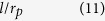	
	3d-I, 3e-I