# Supplementary material for: Nanostructure Formation by controlled dewetting on patterned substrates: A combined theoretical, modeling and experimental study
Source: Sci Rep. 2016 Sep 1;6:32398. doi: 10.1038/srep32398 (PMC5007673; doi:10.1038/srep32398)
Supplement: Supplementary Information [file srep32398-s1.doc]

Nanostructure formation by controlled dewetting on patterned substrates: A combined theoretical, modeling and experimental study

Liang-Xing Lu^1,‡^, Ying-Min Wang^2, ‡^, Bharathi Madurai Srinivasan^1^, Mohamed Asbahi^2^, Joel K. W. Yang^2,3,*^, Yong-Wei Zhang^1^,*

^1^ Institute of High Performance Computing, A*STAR, Singapore 138632, Singapore

^2^ Institute of Materials and Research Engineering, A*STAR, Singapore 117602, Singapore

^3^ Singapore University of Technology and Design, Singapore 138682, Singapore

^‡^ These authors contributed equally.

* Corresponding emails: [joel_yang@sutd.edu.sg](mailto:joel_yang@sutd.edu.sg); [zhangyw@ihpc.a-star.edu.sg](mailto:zhangyw@ihpc.a-star.edu.sg)

**KEYWORDS:**

Solid state dewetting; phase field simulation; nanofabrication; template-assisted

1. **Phase field model**

In our phase field model, three conserved order parameters: , and are employed to denote the phase distribution, i.e. in the vacuum phase, in the film phase and in the patterned substrate phase, with **r** being the position vector of a point, and constraint: in the whole simulation region.

The coarse-grained Ginzburg-Landau free energy of the multiphase system, including bulk free energy, surface energies and interfacial energy, is modeled using the following equation [58]:

(S1)

where $\alpha_{ij}$ are constants related to the height of double-well potential, $k_{ij}$ are the gradient energy coefficients. The surface/interface energy densities are given as [58]:

$\xi_{ij}=\sqrt{2\alpha_{ij}k_{ij}}/3$ (S2)

To simplify the evolution equation, we assume that the phase field that describes the evolution of substrate is static, i.e., it does not evolve with time. Since , we choose as an independent phase field and its evolution follows the Cahn-Hilliard equation [58]:

(S3)

where, $\tau$ is the time and $M$ is the mobility. Noticing that and , and does not evolve with time, we have:

(S4)

Where:

(S5)

1. **Algorithm**

Equation (S3) is solved in the following non-dimensional form:

(S6)

(S7)

where$\tau^{*}=\tau\cdot f_{0}M/\Delta x^{2}$, $L^{*}=L\cdot\Delta x^{2}/M$, $\alpha_{ij}^{*}=\alpha_{ij}/f_{0}$, $k_{ij}^{*}=k_{ij}/f_{0}\Delta x^{2}$, , , $\mathbf{r}^{*}=\mathbf{r}/\Delta x$. Here, and are the length scale and energy scale, respectively. Equation (S6) is solved using Fourier-Spectral method with $\Delta x^{*}=\Delta x/\Delta x\equiv1$ and $\Delta\tau^{*}=0.2$:

(S8)

(S9)

We list below in Table S1 the parameters used for the dewetting simulation. The wetting angle is determined by Equation (S2) and Young’s equation. Four different wetting angles are used in our simulation. For the simulation of Au film on the Si substrate, we use a wetting angle of $\theta\approx140^{\mathrm{o}}$ which is within the range of reported values for the wetting angle of gold on silicon with oxide layer at 400^o^C [34].

Table S1. Parameters used in phase field simulation

| **Free energy constants** | **Value** | |
| --- | --- | --- |
| $\alpha_{ij}^{*}$ | 1 | |
| **Gradient energy coefficients** |  | |
| $k_{12}^{*}=k_{13}^{*}$ | 0.5 | |
| $k_{23}^{*}$ | $\theta=\pi/6$ | 0.01 |
|  | $\theta=\pi/3$ | 0.1 |
|  | $\theta=120^{\mathrm{o}}$ | 1.1 |
|  | $\theta=140^{\mathrm{o}}$ | 1.6 |

1. **Simulation setup**

Before executing the phase field main routine, we first generate the patterned Si substrate and the as-deposited metal film according to given geometry parameters including: width of the mesas $w$, width of the pits $l$, period of the substrate $p=w+l$, height of the mesas $h$ and thickness of the metal film $a$. It should be noted that the geometry generated here is not smooth but in a discrete manner with grid size $\Delta x$, so that we have the non-dimensional parameters of: $p^{*}=\left\langle p/\Delta x\right\rangle$, $w^{*}=\left\langle w/\Delta x\right\rangle$, $h^{*}=\left\langle h/\Delta x\right\rangle$ and $a^{*}=\left\langle a/\Delta x\right\rangle$, where denotes the rounding to the corresponding value.

By comparing with experiments, we find that for Au on Si substrates at 400^o^C, the non-dimensional time is $\tau^{*}\approx22\cdot\tau\cdot\left(p^{*}/p\right)^{2}$. In our phase field simulations, before the start of annealing, we impose an initial perturbation of one grid size to the surface of metal film to consider the surface roughness effect on dewetting process. After the generation of the initial geometry, phase field routine will then be executed based on the model and parameters described above.

**S4. Influence of pit’s depth on equilibrium morphology**

In addition to the deep pit analysis in the main manuscript, here we discuss the influence of shallow pit on the equilibrium.

**Case 1:** $h>a$ and $\theta<\pi/2$

We have shown in our energetic analysis that for a small wetting angle, the film is always confined to the pits. Figure S1 shows the change of equilibrium morphology with increasing the pit depth $h$, starting from {pit|thin} morphology as shown in Figure S1-a. If $h$ is smaller than a critical value of $h_{c1}$, the two wedges are pinned by the mesa corners as shown in Figure S1-b. In this case, the two corner wedges are still separated from each other and the contact angle is . When $h$ is further increased to another critical depth $h_{c2}$, the contact and merge of the two corner wedges takes place as shown in Figure S1-c. Below $h_{c2}$, the contact angle increases with the decrease of the pit depth as shown from Figure S1-c to Figure S1-d. Once the contact angle reaches , the film starts to spill out of the pits and extends onto the adjacent mesas as shown in Figure S1-e. The critical depth corresponding to the spill-over is $h_{c3}$. Below $h_{c3}$, the wetted area of mesas increases with the decrease of $h$, and finally becomes fully wetted at a critical depth of $h_{c4}$ as shown in Figure S1-f, i.e., which corresponds to the pure Wenzel state.

By comparing the morphologies listed in Figure S1 with those in [46], we find that the morphology in Figure S1-a corresponds to the cW and cD phases for $\theta<\pi/4$ and $\theta>\pi/4$, respectively. The morphology in Figure S1-b corresponds to the pW and pD phases, the morphologies in Figure S1-c and Figure S1-d correspond to the F phase, and the morphology in Figure S1-e corresponds to the D phase in [46]. It is noted that no pure Wenzel state is present in [46].

The values of the critical depths of $h_{c1}$, $h_{c2}$, $h_{c3}$, and $h_{c4}$ can be calculated geometrically with constant volume constrains. Usually, they are functions of film thickness $a$, pit width $l$ and wetting angle $\theta$. Depending on the values of these quantities, some of the morphologies may not appear. For example, for {pit|thick}, the morphologies shown in Figure S1-a and S1-b do not appear. In fact, it directly starts from S1-c.


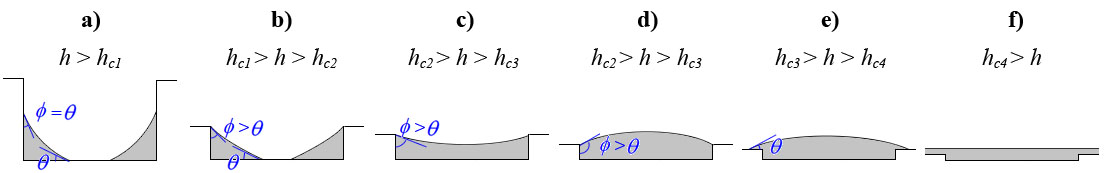


Figure S1. A series of equilibrium morphologies upon the decrease in the pit depth for $\theta<\pi/2$.

**Case 2:** $h>a$, $\theta\geq\pi/2$ and {pit|thin}

For large wetting angle and thin film thickness, the film can also be confined to the pits. But the morphology is different from that for a small wetting angle. As shown in Fig.S2, the film in the pit forms a single nanowire, which is detached from the pit sidewalls. For this case, decreasing the pit depth will not affect the equilibrium morphology as long as $h>a$.


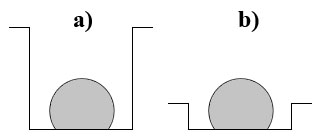


Figure S2. Equilibrium morphology remains unchanged with the pit depth for $\theta\geq\pi/2$ and {pit|thin} morphology.

**Case 2:** $h>a$, $\theta\geq\pi/2$ and {pit|thick}

In this case, the film in the pit is connected the pit sidewalls as shown in Figure S3-a. When the depth decreases to a critical value of $h_{c1}$, the wetting front starts to be pinned by the corners of mesa as shown in Figure S3-b. In this case, the contact angle increases with the decrease of the pit depth and varies in the range of as shown in Figure S3-b and S3-c. Once the contact angle reaches the upper limit , the wetting front starts to de-pin from the corners and spill onto mesas as shown in Figure S3-d. The critical depth corresponding to the spill-over is $h_{c2}$. Below $h_{c2}$, the wetted area of mesas increases with the decrease of the pit depth, and finally becomes fully wetted at a critical depth of $h_{c3}$. Unlike the small wetting angle case, for $\theta\geq\pi/2$, there are two possible morphologies below $h_{c3}$, i.e. Wenzel state and Cassie-Baxter state as shown in Figure S3-e and S3-f, respectively. By a simple energetic analysis, we show that in case of $-\cos(\theta)<1/(1+2X)$, the Wenzel state is energetically favourable, and the Cassie-Baxter (CB) state is favourable when $-\cos(\theta)>1/(1+2X)$, where $X$ is the aspect ratio of the pit depth vs. the pit width.


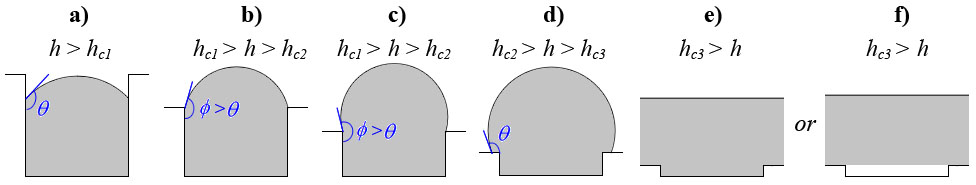


Figure S3. A series of equilibrium morphologies upon the increase in the pit depth for $\theta\geq\pi/2$ and {pit|thick} morphology.

**Case 3:** $h\leq a$

When the pit depth is smaller than the film thickness, the film on mesas and pits are connected with each other even before dewetting as shown in Figure S4-a. In this case, pure Wenzel state or CB state will be formed based on the same criterion as in Case 2, i.e., $-\cos(\theta)<1/(1+2X)$ for the Wenzel state, and $-\cos(\theta)>1/(1+2X)$ for the CB state.


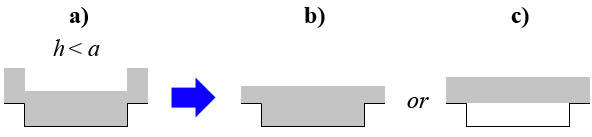


Figure S4. Equilibrium morphology in case of depth smaller then film thickness: $h<a$

**S5. Film deposited on a single mesa: energetic analysis of Regime III**

In case of thick film deposited on a single mesa, there is a possible configuration that the film not only covers the mesa’s top surface but also covers part of the side walls as shown in Figure S5-b. For this configuration, we need to solve the most stable penetration depth $h_{1}$ by energetic analysis. The conserved film volume and the total surface/interface energy for this configuration are expressed, respectively, as:

$V=a\cdot w=r_{m}^{2}\left[\pi-\mathrm{asin}(\frac{w}{2r_{m}})\right]+\frac{w}{4}\sqrt{4r_{m}^{2}-w^{2}}-w\cdot h_{1}$ (S10)

$E_{tot}=\xi_{13}\cdot S_{13}^{0}+2r_{m}\left[\pi-\mathrm{asin}(\frac{w}{2r_{m}})\right]-(2h_{1}+w)\cdot\cos(\theta)$ (S11)

According to Equation (S10) and (S11), the derivative of the total energy with respect to the penetration depth is given by:

$\frac{dE_{tot}}{dh_{1}}=\frac{w}{r_{m}}-2\cos(\theta)$ (S12)

According to Equation (S12), in case of $\theta\geq\pi\text{/2}$, it is always have $dE_{tot}/dh_{1}>0$, which means that in this case the film will always stable on the top of the mesa. However, in case of $\theta<\pi\text{/2}$, there is a critical radius, i.e.:

$r_{mc}=w/2\cos(\theta)$ (S13)

The film is stable on top of the mesa in case of $r_{m}\leq r_{mc}$, but starts to slip down in case of $r_{m}>r_{mc}$. Noticing that the radius of $r_{m}$ increases monotonically with the increase of $h_{1}$, the critical radius in Equation (S13) is actually a stable/unstable criterion: in case of $\theta<\pi\text{/2}$ and $r_{m}>r_{mc}$ (or equivalently $a_{m}>a_{m2}$), the film is unstable on top of the mesa and will “slip down” until the whole film leaves the top. Noticing that before “slip down” (i.e., $h_{1}\text{=0}$), Equation (S13) is equivalent with . So, Equation (S13) also implies that the depinning condition of the contact line from the ridges of the mesa is: .


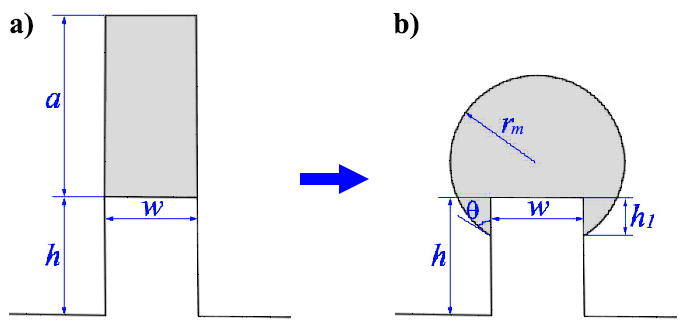


Figure S5. Stability of film deposited on a single mesa. a) as deposited configuration; b) equilibrium shape with a penetration depth $h_{1}$

58. D. J. Seol, S. Y. Hu, Z. K. Liu and L. Q. Chen, J. Appl. Phys. **98**, 044910 (2005)
